# Eco-Friendly Synchronous Spectrofluorimetric Method for Simultaneous Determination of Remdesivir and Acetyl Salicylic Acid in Spiked Human Plasma

**DOI:** 10.1007/s10895-024-03851-1

**Published:** 2024-08-16

**Authors:** Sohair M. aboelghar, Maha A. Hegazy, Hebatallah A. Wagdy

**Affiliations:** 1 Pharmaceutical Chemistry Department, Faculty of Pharmacy, The British University in Egypt, P.O. Box 43, El-Sherouk City, Cairo 11837 Egypt; 2 Health Research Center of Excellence, Drug Research and Development Group, Faculty of Pharmacy, The British University in Egypt, El-Sherouk City, Egypt; 3 Analytical Chemistry Department, Faculty of Pharmacy, Cairo University, Kasr-El-Aini Street, Cairo, 11562 Egypt; 4 Department of Pharmaceutical Analytical Chemistry, Faculty of Pharmacy, Cairo University, Cairo, Egypt

**Keywords:** Spectrofluorimetry, COVID-19, Remdesivir, Acetyl salicylic acid, Spiked human plasma, Greenness assessment tools

## Abstract

Remdesivir and acetyl salicylic acid are often co-administered medications in the treatment of COVID-19, specifically targeting the viral infection and thromboembolism associated with the condition. Hence, it is essential to establish a technique that enables the concurrent quantification of these pharmaceutical compounds in plasma while also keeping environmentally friendly methods. Accordingly, the aim of this work is to simultaneously determine remdesivir and acetyl salicylic acid through a bioanalytical validated synchronous spectrofluorimetric method with applying principles of green chemistry. Since, the two drugs showed severe overlap after excitation at 242.0 nm, 284.0 nm for remdesivir and acetyl salicylic acid, respectively. The overlap was effectively overcome by using synchronous mode with a wavelength difference (Δλ) of 160.0 nm for remdesivir and 100.0 nm for acetyl salicylic acid. Different parameters have been optimized such as Δλ, solvent, pH and surfactant. A linear calibration was obtained over the concentration range 0.01–4.00 µg/mL for remdesivir and 0.01–3.00 µg/mL for acetyl salicylic acid and the method was precise and accurate. The method was successfully used for the investigation of pharmaceutical formulation and the quantification of the maximum plasma concentration (C_max_) of the two drugs. The method has been evaluated as an excellent green analytical method based on three greenness assessment tools.

**Supplementary Information:**

The online version contains supplementary material available at 10.1007/s10895-024-03851-1.

## Introduction

Monitoring co-administrated drugs in the biological matrices is highly important to ensure the safety and efficacy of medicines. Therefore, the process of developing validated bioanalytical techniques is necessary to monitor concurrently administered drugs in biological fluids. When choosing an appropriate strategy, a number of factors should be taken into consideration, including sensitivity, selectivity, analysis speed, and adherence to green chemistry concepts [1]. One of the commonly used instruments in analysis is the spectrofluorometer. It is a simple, sensitive, and selective device that can measure the analyte at very low concentrations and requires minimal sample pretreatment. The sensitivity is higher than that of absorption spectroscopy, as it measures intensity in a direct way without comparing intensity to a reference. In addition, it is more simple than chromatographic analysis since there is no need for the preparation of mobile phases and steps of conditioning [2]. Moreover, it is an inexpensive technique like high-performance liquid chromatography or mass spectrometry; and it consumes small amounts of chemicals, which is very important in green chemistry [3]. The theory relies on estimating how much fluorescent light is released after being exposed to ultra-violet (UV) or visible light [4, 5].

COVID-19 is a global pandemic disease that has threatened the lives of many people worldwide. It is caused by severe SARS-CoV-2 that was identified in patients in Wuhan City [6]. COVID-19 patients suffered from different symptoms such as fever, cough, dyspnea, headache, dizziness, general weakness, vomiting, and diarrhea. In addition, respiratory symptoms range from mild symptoms to significant hypoxia with acute respiratory distress syndrome [7]. It was reported that since July 2020 that there were more than 12 million confirmed cases and a total of more than 500,000 deaths [8]. Therefore, different therapeutic classes were proposed for virus treatment, such as antivirals, anticoagulants, antibiotics, and anti-inflammatory drugs. The drugs of interest in this research are remdesivir (REM) and acetyl salicylic acid (ASA), as they are co-prescribed in the treatment protocol of COVID-19 [9–13].

**Fig. 1:**
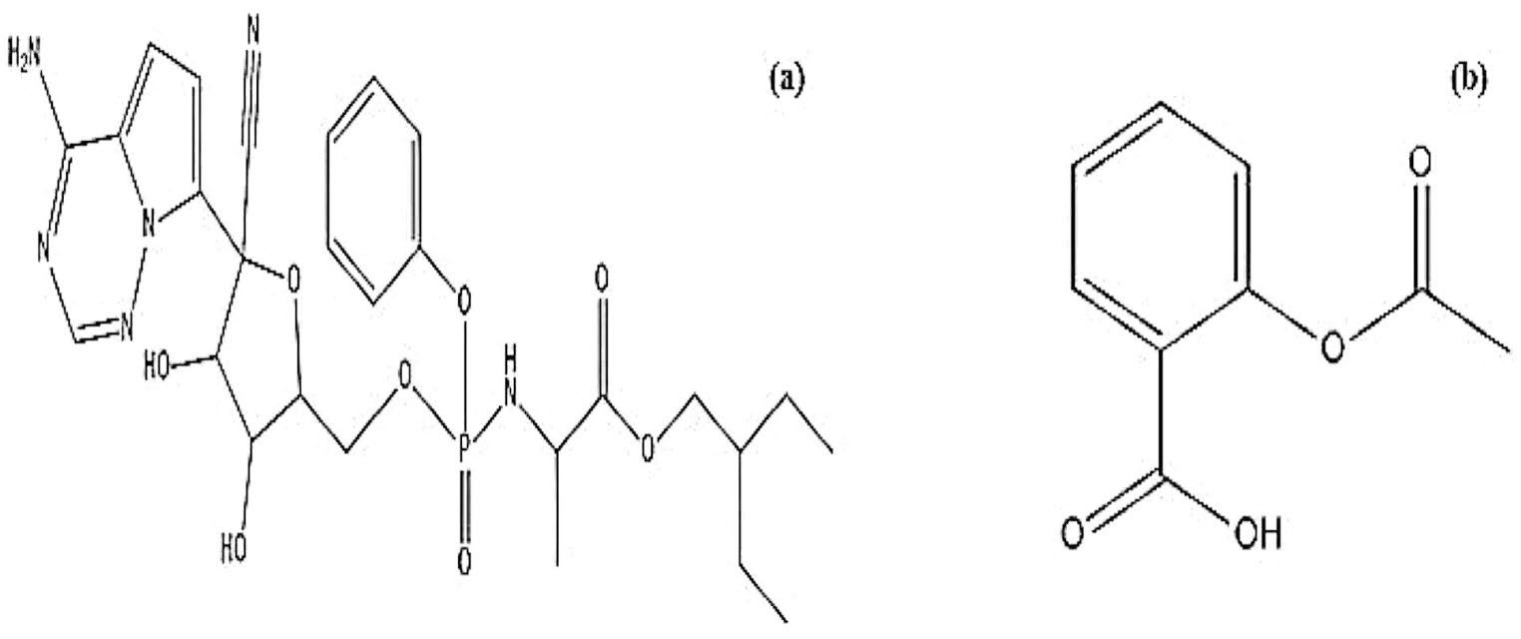
Chemical structure of (**a**) REM, (**b**) ASA

REM (Fig. 1 (a)), chemically known as [(2*R*,3*S*,4*R*,5*R*)-3,4-dihydroxy-5-[4-(hydroxyamino)-2-oxopyrimidin-1-yl] oxolan-2-yl] methyl 2-methylpropanoaint, is a novel antiviral that is used for the treatment of many RNA diseases, including SARS-CoV-2, Middle East respiratory syndrome, and Ebola virus [14, 15]. The FDA has granted REM an emergency use authorization, allowing its use in the COVID-19 outbreak. It is recommended for the treatment of coronavirus disease in adults and children over 12 years old who require hospitalization [16].

REM (GS-5734), a prodrug, undergoes intracellular metabolism to produce an alanine metabolite (GS-704,277), which is converted to a monophosphate derivative and finally to an active nucleoside triphosphate derivative. The active metabolite interferes with RNA-dependent RNA polymerase, which is responsible for viral replication, causing a reduction in viral load [17]. Different analytical techniques were used for the analysis of REM, such as spectrophotometry [18, 19], spectrofluorimetry [20–22], chromatography [23–25] and electrochemical [26].

ASA (Fig. 1 (b)), chemically known as 2-acetyloxybenzoic acid, is an anti-inflammatory and anti-coagulant drug [27]. The drug was repurposed for COVID-19 treatment as clinical trials proved its efficacy in decreasing mortality [13, 28, 29]. The mechanism of ASA depends on targeting the enzymes cyclooxygenase-1 and cyclooxygenase-2 to prevent the body from producing prostaglandins that cause headaches and pain. Moreover, it inhibits platelet aggregation due to interference with thromboxane-A2 in platelets, which in turn inhibits platelet aggregation. Several techniques of analysis were used for the estimation of ASA, such as spectrophotometry [30–34], spectrofluorimetry [35–39], chromatography [40–43], electrochemical [44–47] and proton NMR [48].

Researchers have dedicated their efforts to the advancement of analytical techniques that are environmentally friendly and safer for humans by using solvents and chemicals with low environmental impact, low energy consumption, and lower waste generation [49].

REM and ASA acid are mostly co-prescribed drugs in the treatment of COVID-19 virus, and to our knowledge, no analytical techniques have been developed for their simultaneous determination. Therefore, the objective of this research is to develop and validate a simple, sensitive, synchronous spectrofluorimetric method for the simultaneous determination of REM and ASA in spiked human plasma. The method will be implemented on both the dosage forms and in spiked plasma at the C_max_ of the drugs. Finally, implementing green analytical chemistry concepts.

## Experimental

### Apparatus

- Agilent Cary Eclipse spectrofluorometer was used for all spectral measurements (Agilent, USA). The instrument is equipped with Xenon flash lamp, Czerny-Turner monochromator, red sensitive photomultiplier tube detector and cray eclipse software for recording data. Excitation and emission slit 5 nm and Scan rate 600 nm/min.
- pH Meter: Jenway digital pH- meter 3310 (Dunmow, Essex, United Kingdom) for pH measurements.
- Vortex (VELP Scientifica, Europe) was used for mixing spiked plasma samples.
- Centrifuge (Centurion K241R, UK) was utilized for plasma protein precipitation.

### Materials and Reagents

- REM pure substance with certified purity 99.90% was kindly supplied by Epico pharmaceutical company (Cairo, Egypt).
- ASA pure standard with certified purity 99.90% was purchased from sigma Aldrich company (Dermstadt, Germany).
- REM^®^ vial (100.00 mg/ 20 mL), batch no 2,105,425 and ASA^®^tablets (100 mg), batch no 16vx2/2 were purchased from Egyptian local market.
- Methanol (HPLC grade) was purchased from Fisher Scientific (Loughborough, Leicestershire, UK).
- Analytical grade of Hydrochloric acid was obtained from SD Fine Chem Limited (Mumbai, India).
- Tris base buffer was obtained from Sigma Aldrich company (Dermstadt, Germany).
- Sodium Lauryl Sulfate (SLS), Beta cyclodextrin (β-CD), carboxy methyl cellulose.
- (CMC), cetrimide and tween 80 were purchased from El Nasr pharmaceutical chemicals company (Cairo, Egypt).
- Blank plasma was provided from blood donation bank, Kasr-El-Aini Hospital (Cairo, Egypt).

### Procedures

#### Standard Solutions Preparation

##### Stock and Working Standard Solutions Preparation

Stock standard solutions (1.00 mg/mL) of REM and ASA were prepared by accurately weighing 10.00 mg of each drug and they were transferred into a 10-mL volumetric flask and the volume was completed with methanol. Working standard solutions of REM and ASA (100.00 µg/mL) were prepared by transferring 1 mL from stock solution in a 10-mL volumetric flask and the volume was completed with methanol.

##### Spiked Human Plasma Standard Solutions Preparation

The concentrations used during the optimization of method parameters were as following: 2.00 µg/mL for REM and 3.00 µg/ mL for ASA. The samples were prepared separately by transferring 200 µL from REM working standard solution and 300 µL from ASA working solution in a 10-mL volumetric flask. Afterwards, 1 mL of thawed plasma, 3 mL acetonitrile were added to previously mentioned concentrations and the volume was completed with methanol. The samples were vortexed for 10 min to ensure homogeneity of the sample, then centrifuged for 15 min at 5000 rpm. Aliquots of the upper layer were filtered through 0.22 μm syringe filter. With the same manner, blank samples were prepared but without REM and ASA addition. Afterwards, synchronous mode was adopted.

#### Construction of Calibration Graph and Quality Control Samples

Calibration curve concentrations in spiked human plasma were prepared by transferring 1, 5, 10, 50, 100, 200, 400 µL for REM and 1, 5, 10, 50, 100, 200, 300 µL for ASA into a series of 10 mL volumetric flasks to obtain a final concentration 0.01, 0.05 0.10, 0.50, 1.00, 2.00, 4.00 µg/mL for REM and 0.01, 0.05 0.10, 0.50, 1.00, 2.00, 3.00 µg/mL for ASA. The same procedure illustrated in “Spiked Human Plasma Standard Solutions Preparation” was followed.

Quality control samples were measured with the same way mentioned in “Spiked Human Plasma Standard Solutions Preparation” and concentrations included were in calibration range. LLQC (Lower limit of quantification), Low quantification limit (LQC), medium quality control (MQC), high quality control (HQC) concentrations for REM were 0.05, 1.00, 3.00, 4.00 µg/mL while, ASA concentrations were 0.05, 0.10, 1.00, 3.00 µg/mL for LLQC, LQC, MQC, HQC, respectively.

#### Method Validation

The suggested method was validated according to FDA guidelines with respect to linearity, accuracy, precision, extraction recovery and selectivity [50].

##### Linearity

Linearity was evaluated by analysis of seven different concentrations. The fluorescence spectra were recorded under previously mentioned conditions. A calibration curve was constructed by plotting concentration against fluorescence intensity. Regression equation and validation parameters were calculated.

##### Accuracy and Precision

Accuracy indicates how much the results are near the true value. It was assessed by analyzing 0.05, 1.00, 3.00, 4.00 µg/mL for REM and 0.05, 0.10, 1.00, 3.00 µg/mL for ASA. Each concentration was injected three times within the same day and within different days. Fluorescence intensity was recorded, and percentage recovery was evaluated.

Intraday and interday precision was estimated to ensure the repeatability of the method. Four concentrations 0.05, 1.00, 3.00, 4.00 µg/mL of REM were used for determination of intraday and interday precision. While the concentrations of ASA were 0.05, 0.10, 1.00, 3.00 µg/mL. For intraday precision evaluation, each concentration was injected in triplicates on the same day. As for interday precision each concentration was injected in triplicates on three different days. Percentage relative standard deviation was used to analyze the results.

##### Extraction Recovery

It was measured using LLQC, LQC, MQC, HQC. The following concentrations were used; (0.05, 1.00, 3.00, 4.00 µg/mL) for REM and (0.05, 0.10, 1.00, 3.00 µg/mL) for ASA. The procedure of preparation as mentioned in “Spiked Human Plasma Standard Solutions Preparation”. It was calculated by the following equation.

Extraction recovery = $$\:\frac{Mean\:peak\:area\:of\:drug\:in\:plasma}{Mean\:peak\:of\:drug\:in\:methamol}\:\times\:100$$.

It was evaluated by calculating % recovery and SD.

##### Selectivity

The selectivity of the method was confirmed by analysis of variable ratios of simulated synthetic laboratory mixture pure standard. The concentrations included were 0.05:0.05, 1.00:1.00, 2.00:1.00, 1.00:2.00, 1.5.00:1.00 µg/mL for REM and ASA, respectively. The preparation procedure was the same as mentioned in “Spiked Human Plasma Standard Solutions Preparation”. It was evaluated by % recovery and standard deviation.

#### Application of the Proposed Method

##### Determination of REM and ASA in their Pharmaceutical Formulation in Plasma

REM vial (100.00 mg/ 20 mL) contents were used to prepare various concentrations. Volume equivalent to 10.00 mg was transferred to a 100 mL volumetric flask and volume was completed with methanol to obtain final concentration 100.00 µg/mL. Then, different ratios were prepared from stock solution. The ratios included were the same as synthetic mixture. The process stated in “Spiked Human Plasma Standard Solutions Preparation” was adopted.

Five tablets of ASA were weighted, crushed, and mixed well. Into a 100 mL volumetric flask, the exact amount of powder equivalent to 10.00 mg was transferred to 100 mL volumetric flask and the volume was completed with methanol to obtain final stock concentration 100.00 µg/mL. Stock solution was used for preparation of various ratios. Included ratios matched those of synthetic mixture. The procedure mentioned in “Spiked Human Plasma Standard Solutions Preparation” was followed.

##### Application on C_max_ Level of the Two Drugs

The mixture of REM and ASA in spiked human plasma was prepared at their respective C_max_ values. Aliquots from working standard solution were used for preparation of mixture concentrations; (2.00 µg/mL for REM, 0.50 µg/mL for ASA). The prior concentrations were prepared by transferring 200 µL from REM and 500 µL from ASA from their working solution in a 10 mL volumetric flask; afterwards the steps outlined in “Spiked Human Plasma Standard Solutions Preparation” were carried out.

## Results and Discussion

**Fig. 2:**
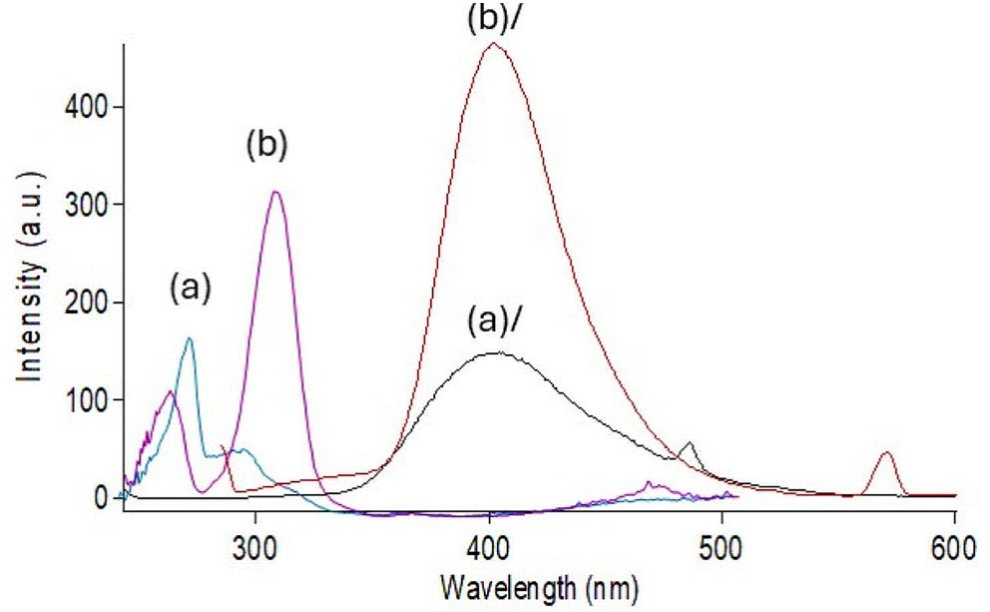
Overlaid Excitation and emission fluorescence spectra of REM (2.00 µg/mL) (**a**, **a/**), ASA (1.50 µg/mL) (**b**, **b/**)

Research reports showed that REM has a significant role in COVID-19 therapy as it decreases the viral load, hospitalization time, and mechanical ventilation progression [51, 52]. Also, various studies discussed how anticoagulants such as ASA have an essential role in reducing thromboembolism and preventing thromboembolic complications in COVID-19 patients [53, 54]. As REM and ASA are co-prescribed together in the treatment and management of COVID-19 disease, their concurrent determination has a great value to be further applied in therapeutic drug monitoring. Consequently, spectrofluorimetry, which is one of the most sensitive analytical techniques, was employed for the estimation of the concentrations of the two drugs. REM and ASA exhibit native emission at 400.0 and 405.0 nm after excitation at 242.0 and 284.0 nm, respectively, as shown in (Fig. 2). The two drugs showed severe overlap after excitation with the mentioned excitation wavelengths, as shown in (Fig. S.1). The first, second, and third-order derivative derivatization techniques were tried to solve the overlapped spectrum, but none of the derivatives were able to solve the overlapped spectrum. Therefore, synchronous mode, which relies on decreasing spectral bands, was adopted [55]. Some variables such as Δλ, diluting solvent, pH, and surfactant, which affect the spectral intensities of the drugs, were optimized, and will be discussed in the upcoming section. The cited drugs were measured at synchronous mode at Δλ = 160.0 nm for REM and Δλ = 100.0 nm for ASA. Fluorescence intensity was recorded, and the drugs were measured at wavelength 230.0 nm for REM and 305.0 nm for ASA.

### Experimental Conditions Optimization

To attain high method sensitivity and resolution, various parameters have been optimized including solvents, pH, and surfactants. Fixed concentrations were used for the evaluation of the mentioned factors. During spectral measurements, one factor was altered while the others remained the same.

#### Δλ Optimization

The value of Δλ has a substantial effect on synchronous fluorescence properties, including resolution of peaks and sensitivity. A wide range of wavelengths Δλ (20.0–200.0 nm) of synchronic spectra were tried to determine the ideal wavelength that will be able to separate the two drugs with high resolution and high sensitivity.

It was found that the best Δλ for REM was 160.0 nm and for ASA 100.0 nm, as they provided narrow peaks with acceptable sensitivity and resolution. For both drugs, synchronous fluorescence intensity decreased while decreasing or increasing specified values.

#### Effect of Diluting Solvents

The effects of different solvents were investigated, including distilled water, methanol, acetonitrile, and acetone. REM and ASA were prepared in a 10 mL volumetric flask by transferring 200 µL for REM, 300 µL for ASA from each working solution separately, then adding 1 mL of plasma, 3 mL acetonitrile, and completing the rest of the volume with the previously mentioned solvents. The same procedure mentioned in “Spiked Human Plasma Standard Solutions Preparation” was followed.

**Fig. 3:**
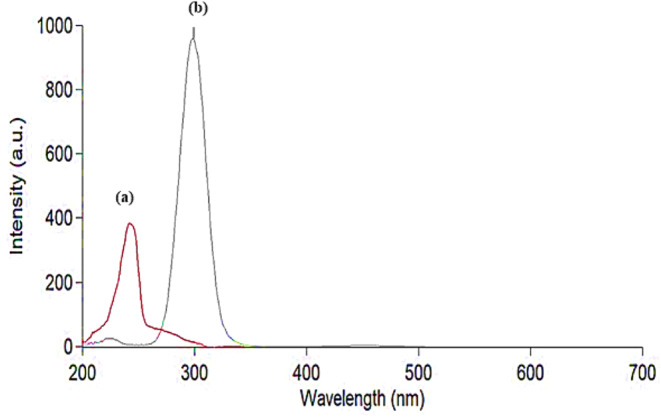
Synchronous spectrofluorimetric determination of (**a**) REM (4.00 µg/mL) at Δλ = 160.0 nm and (**b**) ASA (3.00 µg/mL) at Δλ = 100.0 nm using methanol as solvent

High synchronous intensities were achieved using water, methanol, and acetonitrile for REM, while methanol and acetonitrile produced high intensities for ASA. Acetone decreased the fluorescence intensity greatly in both drugs; thus, it was not used in the experiment. This could be explained by the fact that the polarity of solvents affects fluorescence intensity [56]. The solvent of choice was methanol because it gave the highest intensity for REM and ASA and was consistent with greenness assessment tools. Results are shown in Fig. 3.

#### Effect of pH

The ionization state of the drugs is affected by the pH of the dilution medium, which influences the fluorescence quantum yield [2]. Hence, the pH of the media was investigated. Two buffer solutions were involved: 0.10 M phosphate buffer to cover the acidic range (2.00, 4.00, 6.00) and 0.10 M tris base buffer to cover the basic range (8.00, 10.00, 12.00). REM and ASA were prepared by transferring 200 µL and 300 µL respectively, from their working solution, then 1 mL of buffer with different pH values, 1 mL of plasma, and 3 mL of acetonitrile were added, and the volume was completed with methanol till the mark. The previous process stated in “Spiked Human Plasma Standard Solutions Preparation” was applied.

**Fig. 4:**
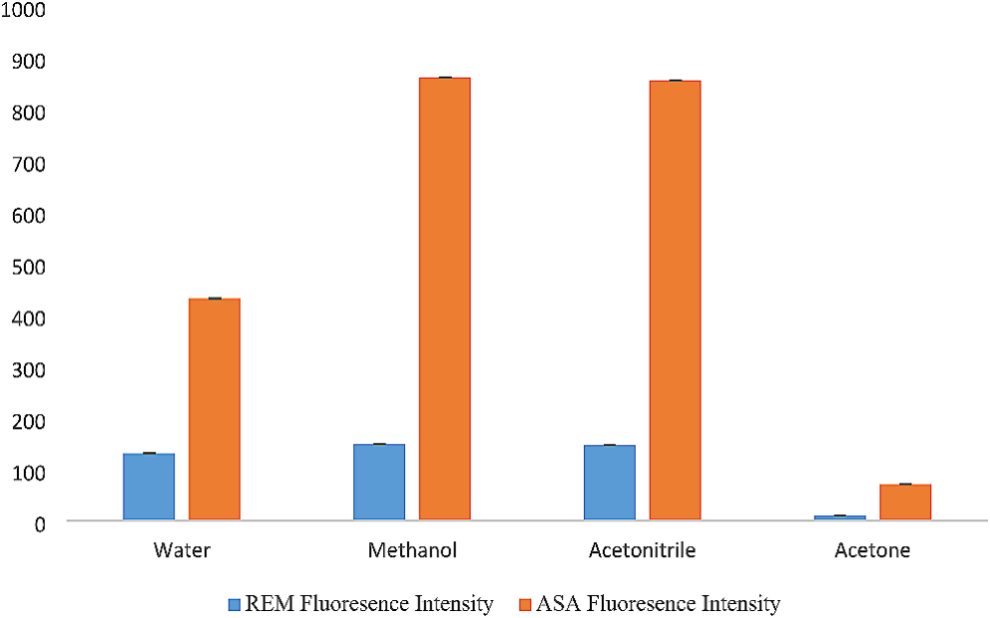
Effect of different solvents on synchronous fluorescence intensity of REM (2.00 µg/mL) and ASA (3.00 µg/ mL)

The spectrum intensities showed an increase in the basic pH conditions, but a reduction in spectral intensities was observed in the acidic range for REM. Regarding ASA, neither acidic nor basic media improved fluorescence intensity. Therefore, the method was completed without buffer addition. The effect of pH on fluorescence intensity is shown in Fig. 4.

#### Effect of Surfactants

Surface active agent addition may change fluorescence quantum yield [2]. Therefore, different surfactants above their critical concentrations were studied, including nonionic, ionic, and cationic surfactants, including Sodium Lauryl Sulfate (SLS), Beta cyclodextrin (β -CD), carboxy methyl cellulose (CMC), Cetrimide, and Tween 80 (at concentrations of 1% w/v). REM and ASA flasks were prepared by transferring 200 µL from their working solution into a 10 mL volumetric flask, then 1 mL of surfactant, 1 mL of plasma, and 3 mL of acetonitrile, and the volume was completed with water. The same method pointed out in “Spiked Human Plasma Standard Solutions Preparation” was accompanied.

**Fig. 5:**
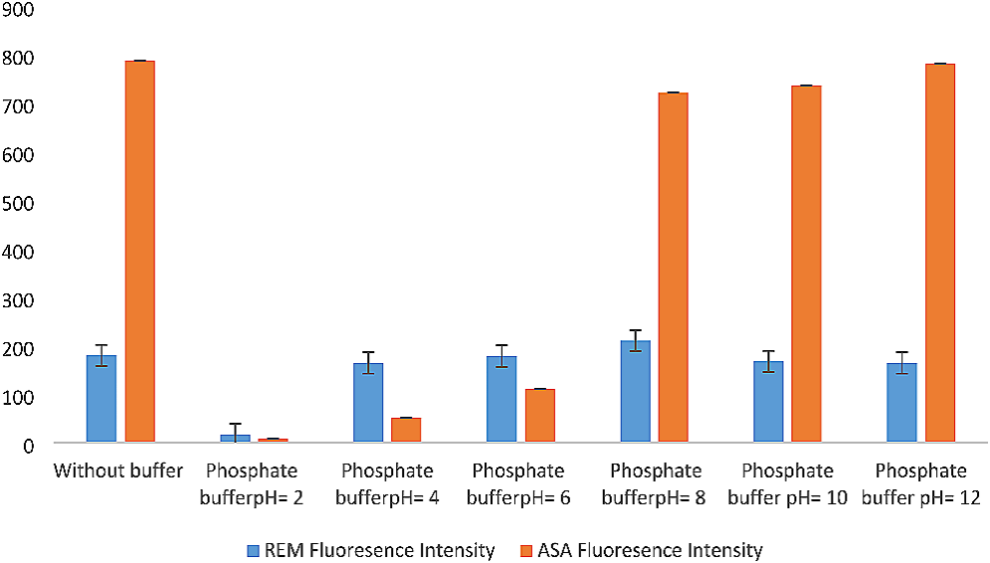
Effect of different pH on synchronous fluorescence intensity of REM (2.00 µg/mL) and ASA (3.00 µg/ mL)

As shown in Fig. 5, for both drugs, there was a negative effect of these surfactants on the fluorescence intensity, as the intensity decreased greatly; therefore, it was not included in the method. The results can be interpreted by the bulky nature of the two analytes and their inability to fit inside micelle chambers created by the surfactant [57].

**Fig. 6:**
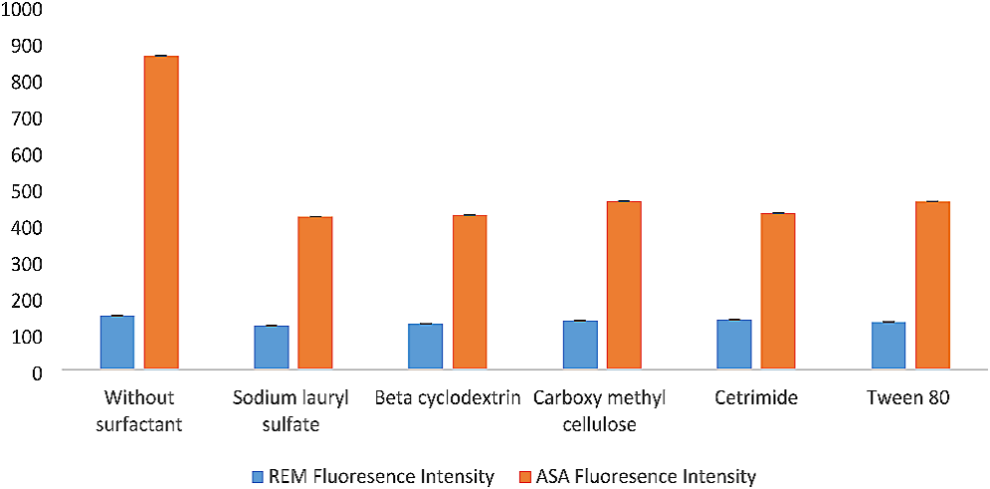
Effect of different surfactants on synchronous fluorescence intensity of REM (2.00 µg/mL) and ASA (3.00 µg/ mL)

Under the optimum conditions illustrated previously, the proposed method was successfully able to separate the two drugs from each other as illustrated in (Fig. 6). REM was estimated at wavelength 230.0 nm and ASA at 305.0 nm.

### Method Validation

The optimized method was validated according to FDA guidelines, as follows:

#### Linearity

**Fig. 7:**
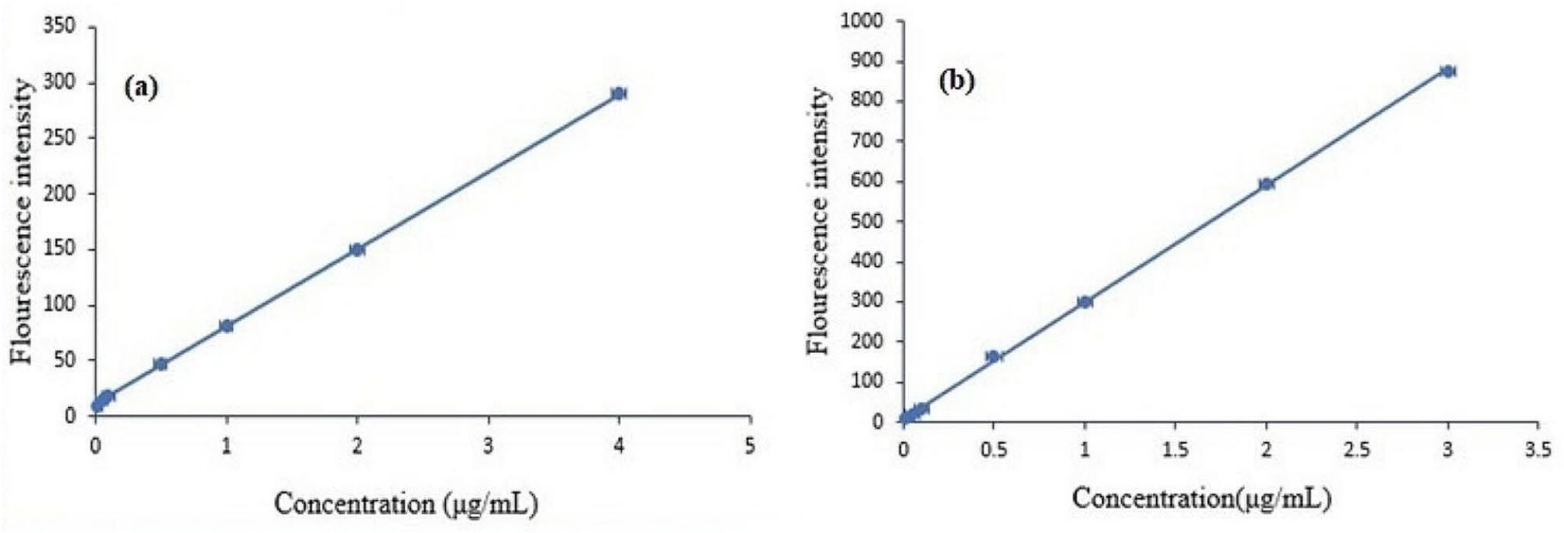
Calibration curve of (**a**) REM from (0.01–4.00 µg/mL) and (**b**) ASA from (0.01–3.00 µg/mL)in spiked human plasma

**Fig. 8:**
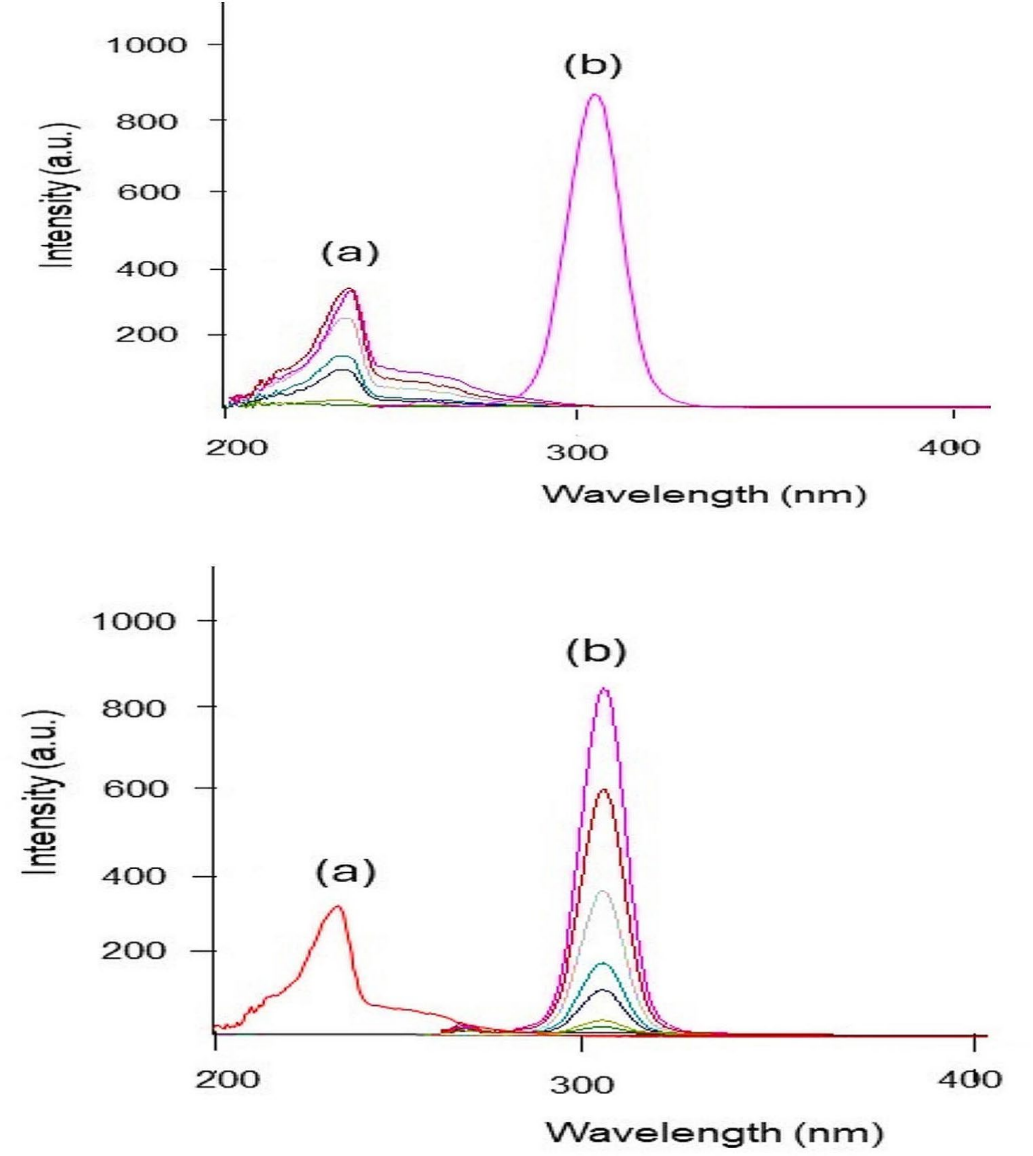
Overlay of different concentrations of (**a**) REM from (0.01–4.00 µg/mL) and (**b**) ASA from (0.01–3.00 µg/mL)

The method was linear over the concentration range of 0.01–4.00 µg/mL for REM and 0.01–3.00 µg/mL for ASA, as illustrated in Figs. 7 and 8, respectively. Regression and validation data are illustrated in Table 1. The high value of R^2^ confirms the linearity as it is near 1, as the accepted criteria for linearity was near to 1 and each concentration within ± 15% of nominal value with exception of LLOQ; accepted limit ± 20%.

**Table 1:** Validation parameters for REM and ASA by the proposed method

Parameters	REM	ASA
Wavelength (nm)	230.0	305.0
Linearity range (µg/mL)	0.01-4.00	0.01-3.00
Slope	70.143	290.47
Intercept (a)	10.962	8.8981
Correlation coefficient	0.9998	0.9997

#### Accuracy and Precision

The method was found to be accurate and precise, as accuracy was confirmed by a high % recovery value and precision was confirmed by a low level of the relative standard deviation (%RSD). % Recovery ranged from 95.97 to 100.25 µg/mL for intraday accuracy, while 99.28-101.58 for interday accuracy. Intraday precision ranged from 0.18 to 1.03 and interday precision ranged from 0.17 to 0.81. Obtained results are shown in Table 2. Accepted range was % recovery ± 15% of nominal concentration and %RSD less than 15% except LLOQ 20%.

**Table 2:** Accuracy, intraday and interday precision of QC samples of spiked human plasma

Concentration (µg/mL) *				Intraday		Interday	
				%Recovery*	%RSD*	%Recovery*	%RSD*
REM	LLQC LQC MQC HQC	0.05	97.41		1.02	97.91	0.78
		1.00	100.94		0.66	99.55	0.60
		3.00	98.71		0.31	98.20	0.58
		4.00	99.59		0.27	99.18	0.45
ASA	LLQC LQC MQC HQC	0.05	96.38		1.03	95.97	0.73
		0.10	99.21		0.71	99.28	0.81
		1.00	98.89		0.21	98.31	0.19
		3.00	101.07		0.18	100.25	0.17

* Average of 3 determinations

LLQC: Lower limit of quantification

LQC: Low quantification limit

MQC: Medium quality control

HQC: High quality control

#### Extraction Recovery

The matrix effect may cause variations in REM and ASA concentrations due to endogenous components of plasma. Therefore, extraction recovery was assessed. The values of % recovery of all samples was more than 94% and the % RSD was less than 2, which demonstrated that no unidentified chemical interfered with the sample matrix. The results are shown in Table 3.

**Table 3:** Extraction recovery by the proposed method

Analyte	Concentration (µg/mL)	% Recovery ± SD*
REM	0.05	94.76 **±** 0.82
	1.00	96.58 **±** 0.49
	3.00	95.88 **±** 0.39
	4.00	96.62 ± 0.27
	Mean ± SD	95.71 ± 0.49
ASA	0.05	94.45 **±** 0.91
	0.10	95.23 **±** 0.87
	1.00	96.74 **±** 0.34
	3.00	96.53 **±** 0.42
	Mean ± SD	94.98 **±** 0.66

*Average of 3 measurements

**Table 4:** Determination of REM and ASA in synthetic mixture by the proposed method

REM		ASA	
Concentration (µg/mL)	%Recovery ± SD	Concentration (µg/mL)	%Recovery ± SD
0.05	97.22 ± 0.22	0.05	96.33 ± 0.33
1.00	99.09 ± 0.32	1.00	98.23 ± 0.19
2.00	98.37 ± 0.15	1.00	97.66 ± 0.22
1.00	98.89 ± 0.37	2.00	98.79 ± 0.34
1.50	99.04 ± 0.56	1.00	99.20 ± 0.41
Mean ± SD	98.52 ± 0.32		98.04 ± 0.29

#### Selectivity

The proposed method was able to determine REM and ASA in synthetic mixture without interference from biological matrix. Choice of these ratios were based on the doses the patient takes in COVID-19 disease. REM is administrated with dose 200.00 mg as a loading dose, then 100.00 mg as maintenance dose [58]. ASA dose from 81.00 to 100.00 mg in COVID-19 proved its efficacy [28, 59]. The results are shown in the Table 4.

### Application of the Method

#### Application to the Dosage Form

The proposed method was successfully applied for the determination of REM and ASA in the pharmaceutical dosage form. The accuracy of the method is illustrated in Table 5.

**Table 5:** Determination of REM and ASA in pharmaceutical formulation by the proposed method

Remdesivir		Acetyl salicylic acid	
Concentration (µg/mL)	%Recovery ± SD	Concentration (µg/mL)	%Recovery ± SD
0.05	96.99 ± 0.32	0.05	95.43 ± 0.53
1.00	98.89 ± 0.41	1.00	97.94 ± 0.21
2.00	97.37 ± 0.22	1.00	97.34 ± 0.33
1.00	98.04 ± 0.61	2.00	98.10 ± 0.62
1.50	97.12 ± 0.74	1.00	98.22 ± 0.28
Mean ± SD	97.68 ± 0.46		97.41 ± 0.39

#### Application of the Method to C_max_ Level of the Two Drugs

The reported C_max_ of REM was 2.28 µg/mL after administration of 150.00 mg [60]. ASA C_max_ 0.54 µg/mL after oral administration of oral 325.00 mg [61]. Hence, a mixture of the two drugs at C_max_ values was prepared. The concentrations included were 2.00 µg/mL for REM and 0.50 µg/mL for ASA.

**Fig. 9:**
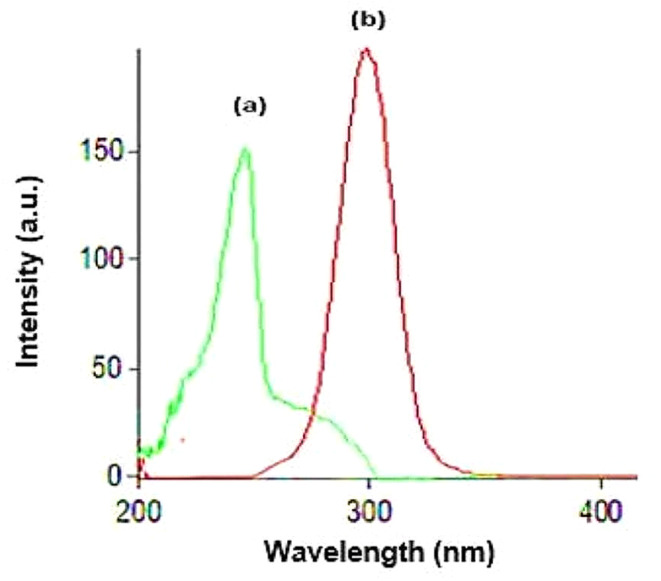
Synchronous spectrum of (**a**) REM (2.00 µg/mL) and (**b**) ASA (0.50 µg/mL) at their C_max_ in spiked human plasma

The suggested method was able to separate REM and ASA in spiked human plasma at their C_max_ values. As a result, the procedure can be used in pharmacokinetic studies and therapeutic drug monitoring in COVID-19 patients. The spectrum of the two drugs at their C_max_ values are shown in Fig. 9.

### Greenness Assessment of the Analytical Method

The reduction of chemical amounts is crucial because it can produce serious risks for people and the environment. Therefore, evaluating the greenness of analytical methodologies is highly important. The concept of green analysis is based on decreasing consumption of the reagents, minimizing energy use, and reducing waste production. The analytical procedure was assessed by two greenness assessment tools: analytical-ecoscale and green analytical procedure index.

#### Analytical Eco-Scale System

An analytical eco-scale system is a tool to assess how environmentally friendly the analytical procedures are. It is based on the deduction of penalty points from 100. Total penalty points are calculated based on the amount of solvent consumed, the hazardous of the instrument, energy consumption, and the amount of waste produced. The penalty points are included in the formula: analytical eco-scale = 100 total penalty points. Calculation results are ranked on a scale; a score > 75 represents excellent green analysis, a score > 50 represents acceptable green analysis, and a score < 50 represents inadequate green analysis [62]. The method is rated as an excellent green method, as shown in Table 6.

**Table 6:** Analytical eco-scale penalty points

Type of reagent	Penalty points
Methanol	(More than 100 mL) = 18
Acetonitrile	4
Hazardousness	(None) = 0
Energy consumption	(Less than or equal to 0.1k W h per sample) = 0
Waste production	3
Total penalty points	25
Analytical Eco-Scale total score	75
Assessment	Excellent green analysis

#### Green Analytical Procedure Index

**Fig. 10:**
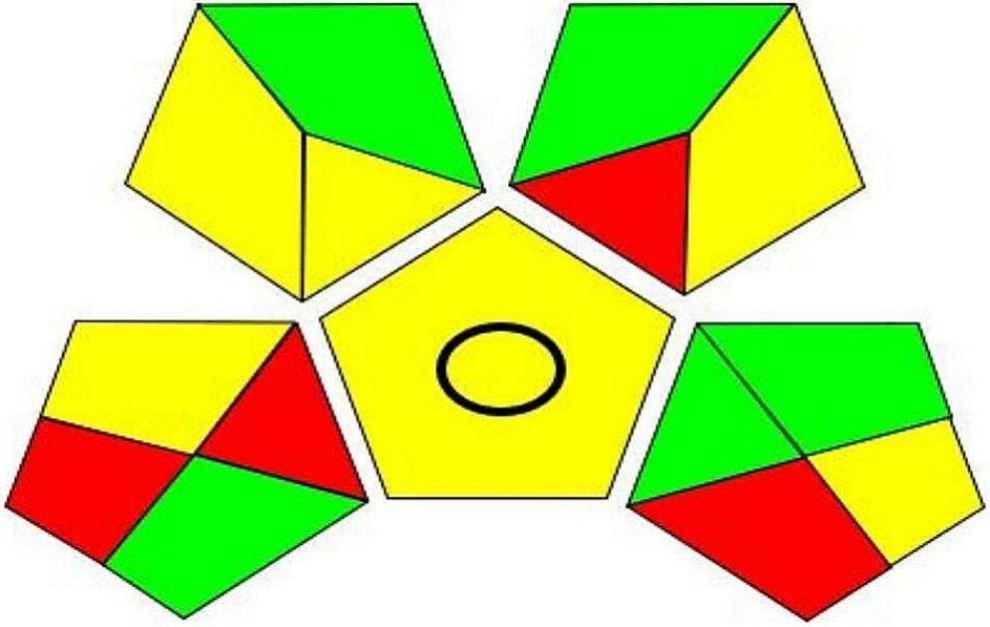
GAPI assessment of the analytical procedure

GAPI is a recent tool for evaluating the greenness of analytical methods. The procedure includes a pictogram representing the sample (collection, preservation, transportation, storage, and preparation), reagents used, and instrumentation. Each pictogram is colored either green, yellow, or red based on the environmental impact [63]. The proposed method showed 7 green regions, 4 yellow regions, and 4 red regions, as illustrated in Fig. 10.

The pictogram showed 5 green regions because of the following reasons: no preservation needed,, no additional treatment for the sample required, no special hazard, instrument energy < 0.1 kWh per sample and analytical procedure hermetically sealed. 6 yellow regions are displayed because of storage under normal conditions, sample preparation with simple procedures, scale of extraction with microscale, green solvents used, and instability score 2. 4 red regions were exhibited because, in sample collection and transportation, the production unit and quality control lab were not in the same place, amounts of solvent used were > 100 mL, amount of waste ranges from 1 to 10 mL and there was no treatment for the waste.

#### Analytical Greenness Metric Approach (AGREE)

**Fig. 11:**
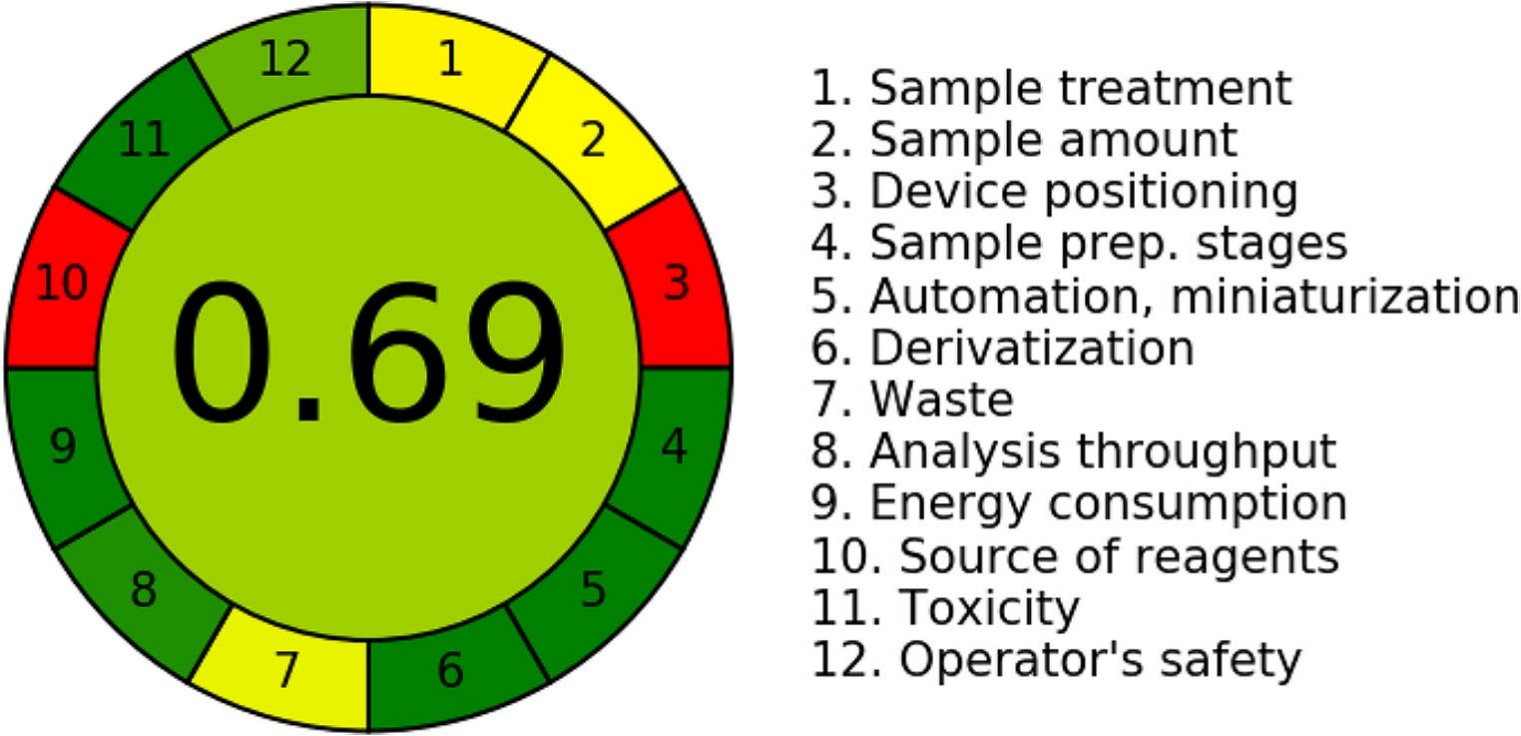
Greenness assessment of the analytical method by AGREE tool

AGREE is a new metric system for assessing the greenness of analytical chemistry procedures. It is based on 12 green analytical chemistry principles, allows assigning different weights to each criteria, provides score on 0–1 scale for each principle and generate a visual clockwise pictogram [64]. The pictogram is shown in Fig. 11. The method achieved an overall score of 0.69 with a green colour, indicating that it is highly green and has low ecological impact.

## Conclusion

A simple, green, sensitive synchronous spectrofluorimetric method has been developed for the quantification of REM and ASA in spiked human plasma, pharmaceutical formulations, and C_max_. The method is eco-friendly and fast, as it does not require treatment with reagents and does not require the preparation of mobile phases as required by chromatographic analysis. The method was assessed as green by the analytical eco-scale system and the green analytical procedure index.

## Electronic Supplementary Material

Below is the link to the electronic supplementary material.

Supplementary Material 1

## Data Availability

All data generated or analyzed during the study are included in the manuscript.
