# Supplementary material for: Eco-Friendly Synchronous Spectrofluorimetric Method for Simultaneous Determination of Remdesivir and Acetyl Salicylic Acid in Spiked Human Plasma
Source: J Fluoresc. 2024 Aug 16;35(7):5059–70. doi: 10.1007/s10895-024-03851-1 (PMC12325558; doi:10.1007/s10895-024-03851-1)
Supplement: Supplementary file 1 — Supplementary Material 1 [file 10895_2024_3851_MOESM1_ESM.docx]

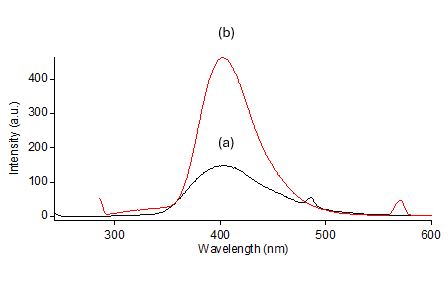


**Fig S.1. Overlay of emission spectra of (a) REM (2.00 μg/mL) and (b) ASA (1.50 μg/mL) after excitation of REM at 242.0 nm and ASA at 284.0 nm.**
